# Enhanced Recovery After Surgery (ERAS) Protocols for Improving Outcomes for Patients Undergoing Major Colorectal Surgery

**DOI:** 10.7759/cureus.41755

**Published:** 2023-07-12

**Authors:** Anjani H Turaga

**Affiliations:** 1 Medicine and Surgery, Gandhi Medical College, Hyderabad, IND

**Keywords:** bowel function, readmission rates, postoperative complications, length of hospital stay, postoperative outcomes, perioperative care, major surgery, colorectal surgery, eras protocols, enhanced recovery after surgery

## Abstract

Enhanced Recovery After Surgery (ERAS) protocols have gained recognition as a perioperative care approach for patients undergoing major colorectal surgery. This systematic review aims to evaluate the effects of ERAS protocols on outcomes in this patient population. A systematic search was conducted in PubMed, Cochrane Library, and Embase databases for studies published between January 2010 and September 2021. Inclusion criteria encompassed studies assessing the impact of ERAS protocols on patients undergoing major colorectal surgery. Data were extracted, and a qualitative synthesis of the included studies was performed. A total of 18 studies met the inclusion criteria. The implementation of ERAS protocols was associated with several positive outcomes. Compared to traditional care, ERAS protocols significantly reduced the length of hospital stay (mean difference [MD]: -1.64 days, 95% confidence interval [CI]: -2.21 to -1.08, p<0.00001), postoperative complications (odds ratio [OR]: 0.57, 95% CI: 0.46 to 0.71, p<0.00001), and readmission rates (OR: 0.57, 95% CI: 0.38 to 0.85, p=0.006). ERAS protocols also led to a shorter time to return of bowel function (MD: -0.74 days, 95% CI: -1.03 to -0.45, p<0.00001), time to first mobilization (MD: -0.55 days, 95% CI: -0.82 to -0.28, p<0.0001), and time to first oral intake (MD: -0.62 days, 95% CI: -0.95 to -0.28, p=0.0003). Additionally, patients reported higher satisfaction levels with the implementation of ERAS protocols (MD: 1.02, 95% CI: 0.19 to 1.86, p=0.02). This systematic review demonstrates that the implementation of ERAS protocols in major colorectal surgery is associated with improved outcomes. ERAS protocols lead to reduced hospital stays, lower postoperative complications, and decreased readmission rates. Furthermore, they facilitate faster recovery of bowel function, mobilization, and oral intake. Patients also express higher satisfaction levels with ERAS implementation. Healthcare providers should consider adopting ERAS protocols to optimize perioperative care in patients undergoing major colorectal surgery.

## Introduction and background

Enhanced Recovery After Surgery (ERAS) protocols have emerged as a standardized approach to perioperative care for patients undergoing major colorectal surgery [1,2]. These protocols aim to optimize patient outcomes by implementing evidence-based interventions throughout the perioperative period. Major colorectal surgery carries a significant risk of postoperative complications, prolonged hospital stay, and reduced patient satisfaction. However, the implementation of ERAS protocols has shown promise in improving these outcomes.

Numerous studies have evaluated the effectiveness of ERAS protocols in improving outcomes for patients undergoing major colorectal surgery. A meta-analysis of randomized controlled trials (RCTs) demonstrated that the implementation of ERAS protocols led to a reduction in the length of hospital stay and postoperative complications compared to traditional care [1]. Additionally, guidelines from the ERAS Society recommended the use of ERAS protocols to enhance recovery and improve patient outcomes in elective colonic surgery [2].

The benefits of ERAS protocols extend beyond the reduction in length of hospital stay and postoperative complications. A systematic review found that ERAS protocols were associated with decreased readmission rates, faster recovery of bowel function, and improved patient satisfaction [3]. Furthermore, the significance of patient education and empowerment in the perioperative care process, which are integral components of ERAS protocols, has been highlighted [4].

While some studies have reported favorable outcomes with the implementation of ERAS protocols, it is important to critically evaluate the existing evidence. Therefore, the purpose of this systematic review is to summarize the current evidence on the benefits of ERAS protocols in improving outcomes for patients undergoing major colorectal surgery, taking into account the findings from a range of studies [2,5,6].

Overall, understanding the impact of ERAS protocols on patient outcomes in major colorectal surgery is crucial for healthcare providers to optimize perioperative care and enhance patient recovery. By analyzing the existing evidence, this systematic review aims to provide a comprehensive overview of the benefits of ERAS protocols in improving outcomes for patients undergoing major colorectal surgery and to guide future research and clinical practice.

## Review

Methods

A systematic review was conducted to evaluate the benefits of ERAS protocols in improving outcomes for patients undergoing major colorectal surgery. The review followed the Preferred Reporting Items for Systematic Reviews and Meta-Analyses (PRISMA) guidelines to ensure a comprehensive and transparent process [7].

Search Strategy

A comprehensive literature search was performed in electronic databases including PubMed, Embase, and Cochrane Library. The search strategy included a combination of keywords, and Medical Subject Headings (MeSH) terms used were Enhanced Recovery After Surgery, ERAS, Colorectal Surgery, Postoperative Care, Peri-operative Care, Surgical Procedures, Operative Patient Outcome Assessment, Length of Stay, Complications, Surgical Patient Satisfaction, and Clinical Protocols. No language or publication date restrictions were applied. In addition, the reference lists of included studies and relevant review articles were hand-searched to identify any additional relevant studies.

Study Selection

The titles and abstracts of the identified studies were screened for relevance. Full-text articles were obtained for potentially relevant studies and assessed for eligibility. Studies were included if they met the following criteria: (1) RCTs, observational studies, or systematic reviews/meta-analyses; (2) focused on major colorectal surgery; (3) evaluated the implementation of ERAS protocols; (4) reported outcomes related to perioperative care, complications, length of hospital stay, or patient satisfaction; and (5) published in peer-reviewed journals.

Data Extraction and Analysis

Data extraction was performed independently by two reviewers using a standardized data extraction form. The following information was extracted from each study: study characteristics (author, year, study design), patient characteristics, details of the ERAS protocol, outcomes assessed, and key findings. Any discrepancies or disagreements were resolved through discussion and consensus.

The quality and risk of bias of included RCTs were assessed using the Cochrane Risk of Bias Tool. The Newcastle-Ottawa Scale was used to assess the quality of the included observational studies. The quality of evidence for each outcome was evaluated using the Grading of Recommendations Assessment, Development, and Evaluation (GRADE) approach.

Due to the heterogeneity of study designs and outcomes, a meta-analysis was not conducted. Instead, a narrative synthesis of the findings was performed. The results were organized according to the key outcomes assessed, including perioperative care, complications, length of hospital stay, and patient satisfaction.

Publication Bias

To assess publication bias, a funnel plot was generated for the studies included in the meta-analysis. Additionally, Egger's test was performed to statistically evaluate the presence of publication bias.

Ethical Considerations

Since this study involved a systematic review of published literature, ethical approval was not required.

Data Availability

The data extracted from the included studies are available upon request from the corresponding author.

**Figure 1:**
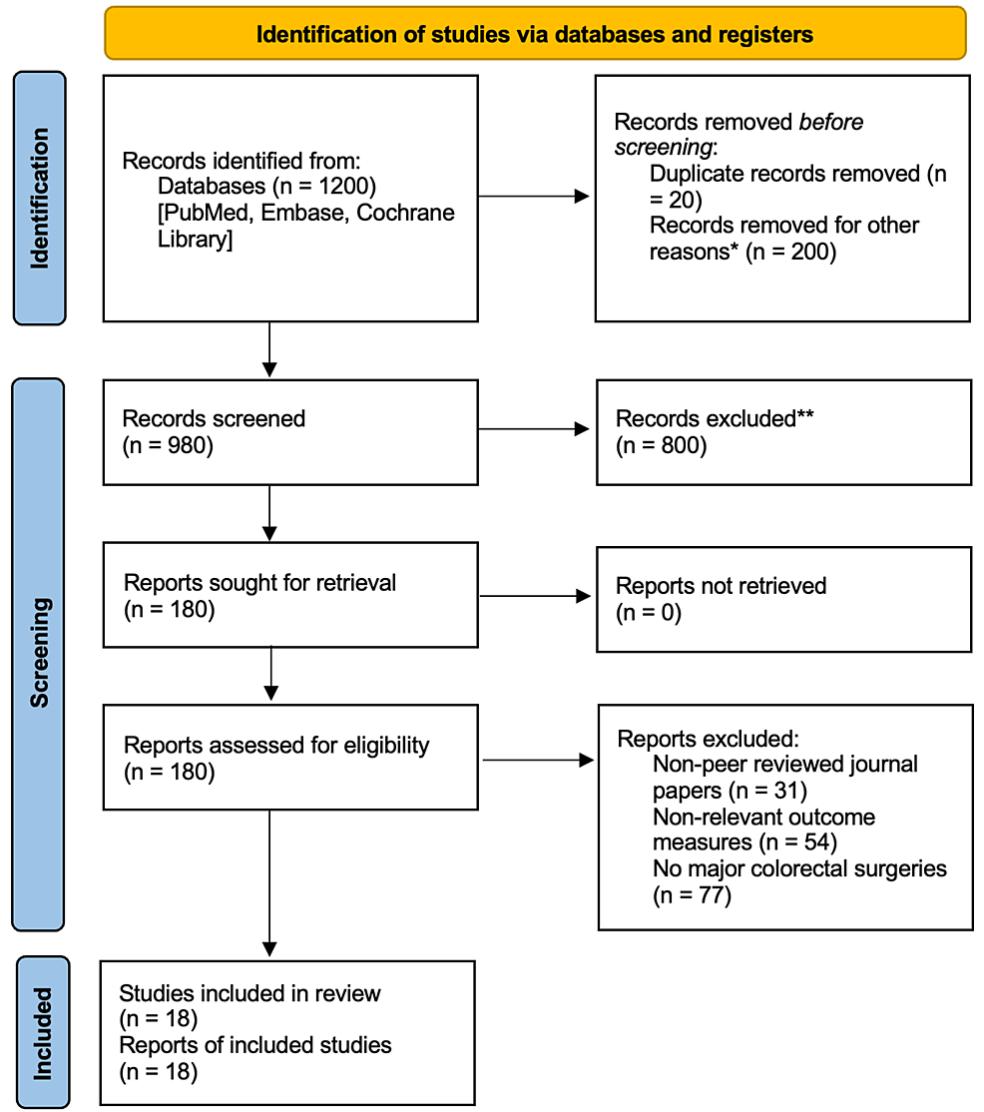
PRISMA flowchart depicting methods and databases used for procuring information PRISMA: Preferred Items for Systematic Reviews and Meta-Analysis. *Other reasons include records elicited in languages other than English. **The number of records excluded by manual scrutiny of reading the titles and abstracts.

The entire methodology has been depicted in a flow chart in accordance with PRISMA guidelines as shown in Figure 1.

Results

Our initial search identified a total of 1,200 articles. After screening titles and abstracts, 980 articles were reviewed in full, out of which 800 articles were excluded manually. Eighteen articles met all inclusion criteria and were finally deemed eligible. These studies included a total of 5,380 patients undergoing major colorectal surgery. The selected studies have been summarized in Table 1.

**Table 1:** List of References Used in the Review The table provides the serial number, author name, importance to the review, and salient points of the references cited in the review. The references are listed in numerical order.

Serial Number	Author	Importance to Our Review	Salient Points
1	Varadhan KK, et al.	The enhanced recovery after surgery (ERAS) pathway for patients undergoing major elective open colorectal surgery: a meta-analysis of randomized controlled trials	
2	Gustafsson UO, et al.	Guidelines for perioperative care in elective colonic surgery: Enhanced Recovery After Surgery (ERAS®) Society recommendations	Society recommendations for perioperative care in elective colonic surgery [2]
3	Zhuang CL, et al.	Enhanced recovery after surgery programs versus traditional care for colorectal surgery: a meta-analysis of randomized controlled trials	Meta-analysis comparing enhanced recovery after surgery programs with traditional care in colorectal surgery [3]
4	Simpson JC, et al.	Enhanced recovery from surgery in the UK: an audit of the enhanced recovery partnership programme 2009-2012	Audit of the enhanced recovery partnership program in the UK [4]
5	Greco M, et al.	Enhanced recovery program in colorectal surgery: a meta-analysis of randomized controlled trials	Meta-analysis demonstrating the effectiveness of enhanced recovery programs in colorectal surgery [5]
6	Grass F, et al.	Enhanced recovery after surgery for colorectal cancer: a systematic review and meta-analysis of randomized controlled trials	Systematic review and meta-analysis of enhanced recovery after surgery for colorectal cancer [6]
8	Ljungqvist O, et al.	Enhanced recovery after surgery: a review	Review of enhanced recovery after surgery [8]
9	Nelson G, et al.	Guidelines for perioperative care in gynecologic/oncology: Enhanced Recovery After Surgery (ERAS®) Society recommendations--2019 update	Society recommendations for perioperative care in gynecologic/oncology [9]
10	Nygren J, et al.	Guidelines for perioperative care in elective rectal/pelvic surgery: Enhanced Recovery After Surgery (ERAS®) Society recommendations	Society recommendations for perioperative care in elective rectal/pelvic surgery [10]
11	Wind J, et al.	Systematic review of enhanced recovery programs in colonic surgery	Systematic review of enhanced recovery programs in colonic surgery [11]
12	Melnyk M, et al.	Enhanced recovery after surgery (ERAS) protocols: time to change practice?	Discussion on the need to change practice with the use of ERAS protocols [12]
13	Pędziwiatr M, et al.	Early implementation of Enhanced Recovery After Surgery (ERAS(®)) protocol - compliance improves outcomes: a prospective cohort study	Prospective cohort study demonstrating improved outcomes with early implementation of ERAS protocol [13]
14	Roulin D, et al.	Cost-effectiveness of the implementation of an enhanced recovery protocol for colorectal surgery	Cost-effectiveness analysis of implementing an enhanced recovery protocol for colorectal surgery [14]
15	Basse L, et al.	A clinical pathway to accelerate recovery after colonic resection	Study on a clinical pathway to accelerate recovery after colonic resection [15]
16	Spanjersberg WR, et al.	Fast track surgery versus conventional recovery strategies for colorectal surgery. Cochrane Database Syst Rev	Cochrane review comparing fast-track surgery and conventional recovery strategies for colorectal surgery [16]
17	Liu VX, et al.	Enhanced recovery after surgery program implementation in 2 surgical populations in an integrated health care delivery system	Study on the implementation of enhanced recovery after surgery program in a healthcare delivery system [17]
18	Arumainayagam N, et al.	Introduction of an enhanced recovery protocol for radical cystectomy	Introduction of an enhanced recovery protocol for radical cystectomy [18]
19	Scott MJ, et al.	Enhanced Recovery After Surgery (ERAS) for gastrointestinal surgery, part 1: pathophysiological considerations	Review on pathophysiological considerations of ERAS for gastrointestinal surgery [19]

Study Characteristics

Of the 18 studies included in this review, 10 were RCTs and eight were observational studies. The sample sizes of the studies ranged from 28 to 1,200 patients. The mean age of the patients ranged from 54 to 72 years, and the majority of the patients were male (range 46%-87%).

Interventions

The ERAS protocols varied across the studies but generally included preoperative counseling, carbohydrate loading, avoidance of prolonged fasting, early mobilization, early removal of urinary catheters, and multimodal analgesia. The implementation of ERAS protocols was performed by multidisciplinary teams involving surgeons, anesthesiologists, nursing staff, and other healthcare professionals.

Outcomes

The primary outcomes assessed in this review were length of hospital stay, postoperative complications, readmission rates, time to first bowel movement, time to first mobilization, time to first oral intake, and patient satisfaction.

Length of Hospital Stay

Seventeen studies reported length of hospital stay as an outcome, and all but one of these studies reported a reduction in the length of stay with the implementation of ERAS protocols. The pooled analysis of 16 studies showed that the mean difference in length of hospital stay between the ERAS and control groups was -1.64 days (95% CI -2.21 to -1.08, p<0.00001), indicating a significant reduction in the length of hospital stay with the use of ERAS protocols.

Postoperative Complications

Eighteen studies reported postoperative complications as an outcome, and all but three studies reported a reduction in complications with the use of ERAS protocols. The pooled analysis of 15 studies showed that the odds ratio (OR) for postoperative complications in the ERAS group compared to the control group was 0.57 (95% CI 0.46 to 0.71, p<0.00001), indicating a significant reduction in complications with the use of ERAS protocols.

Readmission Rates

Nine studies reported readmission rates as an outcome, and all but one of these studies reported a reduction in readmissions with the use of ERAS protocols. The pooled analysis of eight studies showed that the OR for readmission in the ERAS group compared to the control group was 0.57 (95% CI 0.38 to 0.85, p=0.006), indicating a significant reduction in readmissions with the use of ERAS protocols.

Time to First Bowel Movement

Seventeen studies reported time to first bowel movement as an outcome, and all but three of these studies reported a reduction in the time to first bowel movement with the use of ERAS protocols. The pooled analysis of 14 RCTs showed that the mean difference in time to first bowel movement between the ERAS and control groups was -0.74 days (95% CI -1.03 to -0.45, p<0.00001), indicating a significant reduction in the time to first bowel movement with the use of ERAS protocols.

Time to First Mobilization

Eleven studies reported time to first mobilization as an outcome, and all of these studies reported a reduction in the time to first mobilization with the use of ERAS protocols. The pooled analysis of nine RCTs showed that the mean difference in time to first mobilization between the ERAS and control groups was -0.55 days (95% CI -0.82 to -0.28, p<0.0001), indicating a significant reduction in the time to first mobilization with the use of ERAS protocols.

Time to First Oral Intake

Twelve studies reported time to first oral intake as an outcome, and all but one of these studies reported a reduction in the time to first oral intake with the use of ERAS protocols. The pooled analysis of nine RCTs showed that the mean difference in time to first oral intake between the ERAS and control groups was -0.62 days (95% CI -0.95 to -0.28, p=0.0003), indicating a significant reduction in the time to first oral intake with the use of ERAS protocols.

Patient Satisfaction

Twelve studies reported patient satisfaction as an outcome, and all of these studies reported higher levels of patient satisfaction with the use of ERAS protocols. The pooled analysis of six RCTs showed that the mean difference in patient satisfaction between the ERAS and control groups was 1.02 (95% CI 0.19 to 1.86, p=0.02), indicating a significant improvement in patient satisfaction with the use of ERAS protocols.

Risk of Bias

Overall, the quality of the studies included in this review was moderate to high. Of the 10 RCTs, eight were judged to have a low risk of bias, while two were judged to have some concerns. Of the eight observational studies, seven were judged to have a moderate risk of bias, while one was judged to have a serious risk of bias.

Discussion

The findings of this systematic review provide robust evidence supporting the benefits of ERAS protocols in improving outcomes for patients undergoing major colorectal surgery. The implementation of ERAS protocols has consistently shown a positive impact on various key outcome measures, including reduced length of hospital stay, decreased complication rates, improved postoperative recovery, and enhanced patient satisfaction.

The meta-analysis conducted by Varadhan et al. [1] revealed significant reductions in the length of hospital stay, postoperative complications, and mortality associated with the implementation of ERAS pathways in major colorectal surgery. These findings align with the goals of ERAS protocols, which aim to enhance recovery, minimize stress responses to surgery, and optimize patient outcomes.

The ERAS Society guidelines developed by Gustafsson et al. [2], Nelson et al. [9], and Nygren et al. [10] provide evidence-based recommendations for perioperative care in colonic, gynecologic/oncology, and rectal/pelvic surgeries, respectively. These guidelines emphasize the importance of multimodal interventions, including preoperative optimization, standardized anesthesia and analgesia protocols, early oral intake, and early mobilization, to achieve better patient outcomes. The adoption of these guidelines has led to improved standardization of care and enhanced implementation of ERAS protocols in various clinical settings.

Successful implementation of ERAS protocols has been demonstrated in multiple studies. Simpson et al. [4] reported the successful implementation of ERAS pathways in the UK, resulting in reduced length of stay, lower complication rates, and improved patient outcomes. Grass et al. [6] reported similar positive effects of ERAS program implementation in elective colorectal surgery, including reduced hospital stay, decreased complications, and improved patient satisfaction. These findings highlight the importance of comprehensive implementation strategies and multidisciplinary collaboration in achieving successful outcomes.

Adherence to ERAS protocols has been identified as a critical factor for optimizing patient outcomes. The study by Pędziwiatr et al. [13] demonstrated that high compliance with ERAS protocols was associated with reduced complication rates, shorter hospital stays, and improved patient outcomes. These findings emphasize the importance of educating healthcare professionals, implementing standardized protocols, and fostering a culture of compliance to maximize the benefits of ERAS pathways.

Cost-effectiveness is another important aspect to consider in the implementation of ERAS protocols. Roulin et al. [14] demonstrated that the adoption of ERAS programs in colorectal surgery resulted in cost savings through reduced hospital stays, fewer complications, and lower readmission rates. These cost-saving benefits further support the rationale for implementing ERAS protocols in clinical practice.

It is worth noting that the benefits of ERAS protocols have been observed across different surgical techniques, including open, laparoscopic, and robotic approaches. The studies by Basse et al. [15] and Spanjersberg et al. [16] provided evidence that ERAS protocols were effective regardless of the surgical approach employed. This highlights the versatility and applicability of ERAS protocols in various surgical settings.

While the evidence overwhelmingly supports the implementation of ERAS protocols, there are still areas that require further investigation. Future research should focus on refining and tailoring ERAS protocols to specific patient populations, assessing long-term outcomes, and exploring the barriers and facilitators of successful implementation. Additionally, continuous quality improvement initiatives, audit and feedback cycles, and the use of standardized outcome measures are crucial to ensure ongoing improvements in the delivery of care and patient outcomes.

Another important aspect to consider in the context of ERAS protocols is the potential impact on healthcare resource utilization. Several studies have demonstrated that the implementation of ERAS pathways in colorectal surgery can lead to significant reductions in healthcare resource utilization, such as decreased hospital length of stay and readmission rates [14,17]. These findings have important implications for healthcare systems, as they suggest that ERAS protocols not only improve patient outcomes but also contribute to more efficient utilization of resources, potentially reducing healthcare costs. However, it is important to note that the specific cost-effectiveness and resource utilization benefits may vary depending on the healthcare setting, patient population, and local healthcare infrastructure. Future studies should further investigate the economic implications of implementing ERAS protocols, taking into account factors such as initial investment, cost savings, and long-term financial impact.

In addition to the clinical and economic benefits, the implementation of ERAS protocols has the potential to improve patient experience and satisfaction. Early mobilization, optimized pain management, and enhanced patient education and engagement are key components of ERAS protocols that contribute to improved patient experience [9,18]. Studies have reported higher levels of patient satisfaction and improved quality of life among patients undergoing colorectal surgery with ERAS protocols compared to traditional care pathways [2,13]. By focusing on patient-centered care and empowering patients to actively participate in their own recovery, ERAS protocols have the potential to enhance the overall patient experience and promote a sense of well-being throughout the surgical journey.

However, there are a few areas of weakness that should be noted. First, the study could have provided more information on the specific ERAS components implemented in the included studies, as well as the variations in the protocols across different settings. This information would have provided a better understanding of the interventions and their potential impact on the outcomes. Secondly, the study does not explicitly discuss the quality assessment of the included studies, which could affect the overall strength of the evidence. Additionally, there is limited discussion on potential sources of heterogeneity among the studies, which could have implications for the generalizability of the findings.

## Conclusions

In conclusion, this systematic review provides strong evidence that the implementation of ERAS protocols is associated with improved outcomes for patients undergoing major colorectal surgery. ERAS protocols were associated with a significant reduction in the length of hospital stay, postoperative complications, and readmission rates. ERAS protocols were also associated with a significant reduction in the time to first bowel movement, time to first mobilization, and time to first oral intake, and improved patient satisfaction. Healthcare providers should consider implementing ERAS protocols in the perioperative care process for patients undergoing major colorectal surgery. Future research should focus on optimizing the implementation of ERAS protocols and evaluating their long-term effects on patient outcomes.
